# Supplementary material for: Using Machine Learning to Predict Bacteremia in Febrile Children Presented to the Emergency Department
Source: Diagnostics (Basel). 2020 May 15;10(5):307. doi: 10.3390/diagnostics10050307 (PMC7277905; doi:10.3390/diagnostics10050307)
Supplement: Supplementary file 1 [file diagnostics-10-00307-s001.pdf]

Supplemental Table 1. The odds ratio of each variable and its 95%CI after repeating 100 times univariate logistic regression in subgroup study.

Variable	OR	OR (95%CI)	Number of P-value < 0.05
AGE	0.9552	(0.6469 - 1.4026)	0
WBC	0.8965	(0.7656 - 1.0401)	5
CRP	1.0070	(1.0027 - 1.0112)	100
Hemoglobin	0.6378	(0.2347 - 1.9165)	0
MCV	0.9509	(0.3990 - 2.4978)	0
MCH	1.5593	(0.1012 - 18.1577)	0
MCHC	0.9889	(0.1299 - 9.9122)	0
Platelet	0.9998	(0.9973 - 1.0023)	0
Lymphocyte	0.9861	(0.9708 - 1.0016)	24
AST	1.0046	(0.9933 - 1.0152)	2
ALT	0.9991	(0.9835 - 1.0119)	0
RBC	2.4156	(0.1148 - 39.5038)	0
Band	1.0732	(0.9417 - 1.2074)	8
Monocyte	0.9760	(0.9269 - 1.0244)	0
Eosinophil	0.7661	(0.5911 - 0.9558)	84
Basophil	1.4543	(0.6893 - 2.8285)	0
ANC	1.1907	(0.9341 - 1.5174)	9
Segment+Band	0.9694	(0.9427 - 0.9975)	67

WBC, white blood cell; ANC, absolute neutrophil count; MCV, mean corpuscular volume; MCH, mean corpuscular hemoglobin; MCHC, mean corpuscular hemoglobin concentration; ALT, alanine transaminase; AST, aspartate transaminase; CRP, C-reactive protein.

Supplemental Table 2. The maximum value, minimum value, mean value, and standard deviation of multivariate binary logistic regression coefficients of significant risk factors in subgroup study.

Variable	min	-	Max	(	Mean	±	SD	)
CRP	0.0041	-	0.0084	(	0.0062	±	0.0011	)
EOSINOPHIL	-0.2891	-	-0.1597	(	-0.2294	±	0.0284	)
Segment+Band	-0.0481	-	-0.0192	(	-0.0324	±	0.0060	)

CRP, C-reactive protein.
